# Proceedings of the 2019 American Bee Research Conference

**DOI:** 10.3390/insects11020088

**Published:** 2020-01-29

**Authors:** Michael Simone-Finstrom, Elina L. Niño, Michelle L. Flenniken, Hanna Arrowsmith, Judy Wu-Smart

**Affiliations:** 1 American Association of Professional Apiculturists, Lincoln, NE 68583, USA; elnino@ucdavis.edu (E.L.N.); michelle.flenniken@montana.edu (M.L.F.); jwu-smart@UNL.EDU (J.W.-S.); 2 USDA-ARS, Honey Bee Breeding, Genetics and Physiology Research Laboratory, Baton Rouge, LA 70820, USA; 3 University of California ANR Cooperative Extension, Davis, CA 95616, USA; 4 Plant Sciences & Plant Pathology Department, Montana State University, Bozeman, MT 59717, USA; 5 Department of Entomology, University of Nebraska, Lincoln, NE 68583, USA; hannakarrowsmith@gmail.com

**Keywords:** *Apis mellifera*, honey bee biology, apiculture, American Association of Professional Apiculturists

## Abstract

The 2019 American Bee Research Conference (ABRC) was held January 10–12, 2019 in conjunction with the annual convention of the American Honey Producers Association in Tempe, AZ. Over the three-day conference, a total of 45 oral presentations and 13 poster presentations were given, representing work done from over 27 institutions and 34 different research groups from throughout the United States and Canada. This proceedings contains and overview of the conference and the submitted abstracts for presentations given at the 2018 American Bee Research Conference.

## 1. Introduction and Overview

The American Association of Professional Apiculturists (AAPA) organizes an annual American Beekeeping Research Conference (ABRC). In January 2019 the ABRC was held in conjunction with the 50th American Honey Producers Convention and Tradeshow in Phoenix, AZ. AAPA is a professional group of researchers and extension specialist whose main goals are to provide a venue for scientific discussion and rapid dissemination of research findings that will benefit honey bees and beekeepers. Every year we also strive to report on the most up-to-date research findings via print media. We are especially proud of the support that the Association provides to young researchers in training.

The keynote speaker was Dr. Michelle Flenniken (Montana State University) who presented her work on understanding “The impact of viruses on honey bees at the colony, individual, and cellular levels”. This is a topic that is of great interest, especially to beekeepers, as the Varroa mite-virus complex has been implicated in high colony losses across the globe. Flenniken has conducted elegant research involving both longitudinal field studies that have led to discovery of Lake Sinai viruses in association with honey bee colonies and innovative laboratory studies that have enhanced our understanding of the antiviral immune responses of honey bees. Comprehensive studies led by Flenniken are exactly the type of research needed to help further efforts of improving bee health.

This was a great introduction to the talks that followed for which we provide a few highlights below. In the unofficial Honey Bee Stressors session speakers, among other topics, discussed viral spillover to other members of the order Hymenoptera. Alex Payne (TAMU) reintroduced a topic of the detection of honey bee-associated viruses (including DWV) in two of the six tested ant species. For those that have had ants in your hives, it is important to note that these results do not necessarily indicate that ants transmit DWV. Considering current discussions of honey bee colony placements near natural areas and potential implications for native bees, timely research was presented by Samantha Ann Alger (UVM) on the topic of virus transmission between different genera of bees (i.e., honey bee and bumble bee species). Alger detected DWV and BQCV in a bumble bee species and discussed virus circulation within the environment. She has also presented data that viruses can persist on flowers, which can then serve as site of virus transmission (both within and between different bee genera and species).

As it happens in every beekeeping meeting, the discussion quickly turned to Varroa mites, but instead of discussing the potential effects of mites on workers, drones or the entire colony, Lauren Rusert (NCSU) focused her efforts on understanding how Varroa mites might affect queen quality and reproductive success. To date Varroa has not reached the Hawaian islands of Maui, Kauai, Lanai, and Molokai, which was conveniently used to set up mating nucs on islands without Varroa presence and islands where Varroa was introduced in the past decade. The main question was whether queens mating in the absence of Varroa mites have higher mating frequencies. This was not shown to be the case which perhaps is not that surprising since Varroa do not reproduce on developing queens. While mites do reproduce on drones and could potentially affect drone mating success, an area flooded with drones could have a sufficient number of successful matings that would not affect queen reproduction. Rusert detected an average mating frequency of 30 drones and did find variation in sperm count dependent on the island of origin but not correlated with mite presence.

Data indicate that while individual stressors can negatively impact honey bee health, a combination of two or more can significantly exacerbate symptomology. Perhaps the best studied and understood are synergistic effects of various pesticides on honey bee health. One such combination is that of Tilt (a fungicide) and Altacor (an insecticide) which has been shown to be quite toxic to bees in a laboratory setting (E. Walker, OSU). The authors hypothesize that Tilt might inhibit p450s (enzymes that help detoxify pesticides) which prevents Altacor from being broken down causing increased toxicity to bees. A question of whether different genetic stocks might have tolerance to pesticide exposure was tackled by Joe Milone (NCSU) by exposing eight different stocks to various dosages of different compounds. Indeed, Milone presented data that indicated a few of the stocks were more susceptible to pesticide exposure when exposed in larval stages. Sarah Wood (U Saskatchewan) also presented her findings indicating that developing bees might be more resistant to a specific neonicotinoid and a fungicide as compared to exposed adults. Additional presentations explored the impact of various agrochemicals and combinations thereof on both honey bee queen health (E. Walsh, TAMU; I. Kozii, U Saskatchewan) and foraging and recruitment behavior (B. Ohlinger, V Polytechnic Institute and State University).

The final session of the first day of the conference discussed the nutritional needs of the honey bees. Presenters covered topics that included understanding honey bee foraging and pollinator communities in various landscapes such as soybean fields (L. Hearon, OSU) and prairies in the mid-west (M. Carr-Markell, UMN). Pierre Lau (TAMU) presented data addressing the question of whether honey bees search out a specific protein:carbohydrate ratio in plants to satisfy the nutritional needs of the colony. While bee nutrition research has heavily focused on understanding macronutrient needs of honey bees, there is growing evidence indicating that more research should focus on plant micronutrients that might prove very beneficial particularly for developing supplemental feeds for honey bees.

The second day of the conference continued the discussion of various stressors and bees’ physiological responses, as well as a much needed discussion of some more novel tools in our war on Varroa mite. Hongmei Byarlay (Central State University, OH) presented work on the behavioral and anatomical advantages of “ankle-biting” bees which were introduced to the beekeeping world by Greg Hunt and Krispn Given and continue to be bred for by Indiana and other regional beekeepers. Byarlay used 3-D imaging to characterize worker mandibles and found clear differences in shape between “ankle-biters” and non-selected commercial stock. Greg Hunt (Purdue) followed up by reporting that after 11 years of selecting for mite-biting behavior the project now involves beekeepers in seven mid-western states. The research is focused on better characterizing impact on mite levels, but also on viral levels. Thus far, 850 beekeepers have directly participated in extension education and stock sharing through this project. Additionally, Thomas O’Shea-Wheller (LSU) and Michael Simone-Finstrom (USDA-ARS, LA) discussed potential benefits of the new VSH-Pol line stock in terms of resistance/tolerance to various stressors. This stock is not yet available to beekeepers but make sure to stay tuned as it seems like a promising addition.

Many believe that breeding for specific traits is a key long-term solution to many issues facing beekeepers, but we cannot ignore the importance of development of novel miticidal compounds or novel ways of dispensing those already on the market. Edmund Stark (MSU) reported on the recent advancements of the time-controlled release of oxalic and formic acids for mite control. If you have not heard about this project, Stark is a chemist who partnered with bee experts to test the use of a polymer infused with either oxalic or formic acid and this year he reported on some promising results of a field trial. This is exciting news for beekeepers but admittedly there is more work to be done on this system that is not quite yet prime-time ready.

The conference ended with talks addressing several different questions about queen and drone health, environmental stressor effects on bee health, and investigation of potential novel antifungal agents for *Nosema*, which might be of great interest to beekeepers considering the lack of management tools for *Nosema*. Rightfully, there was quite a bit of focus on improving our understanding of various stressors or combination of said stressors on bee health, but there was also exciting work presented on how we might be able to mitigate some of these threats. Research that is being done by Priyadarshini Chakrabarti (OSU) is specifically addressing the impact of phytochemicals on bee health with specific focus on 24-methylenecholesterol, a sterol crucial for bee health. Chakrabarti has found that bees eating this compound had increased abdominal fat and head protein content, and most importantly, they lived longer in cage studies. Future work aims to determine which naturally occurring pollens might contain optimal levels of this compound so that this can be used as part of a management program. 

See below for more specifics and submitted abstracts for presentations from the 2019 ABRC.

## 2. Abstracts of Presentations

### 2.1. Is the Honey Bee Mouth Microbiome Affected by Propolis within the Colony?

DalenbergHollie W.AndersonKirkSpivakMarlaUniversity of Minnesota, Minneapolis, MN, USA

*Apis mellifera* collect tree resins and deposit them within their hive, which is then called propolis. Propolis has been shown to have antimicrobial properties against honey bee pathogens such as American foulbrood (caused by *Paenibacillus larvae*) and chalkbrood (caused by *Ascosphaera apis*) in vitro and in situ. Propolis also has been shown to reduce resting immune system levels in adult bees, possibly allowing for an increased immune response to pathogens. It is unknown if propolis reduces the total microbial load within the colony or if it is active only against specific bacteria or fungi. Honey bees do not eat propolis but they do manipulate it within the colony with their mouthparts. The goal of this research is to determine if exposure to propolis in the nest environment changes the community structure or abundance in the microbiome on honey bee mouthparts. As honey bees use their mouthparts for feeding larvae and oral trophallaxis, revealing the relationship between the antimicrobial properties of propolis and microbial composition on the mouthparts is important for understanding pathogen transmission between bees. After exposure to propolis in field colonies, or in cages in the laboratory, microbiome sequencing will be done on honey bee worker mouthparts that were removed using sterile technique. DNA extraction and sequencing will follow similar techniques in other honey bee mouth part microbiome literature. This research is a first step in understanding how the honey bee microbiome has evolved in relation to constitutive exposure to propolis within the colony, and possible effects on honey bee health when propolis is lacking in the hive.

### 2.2. Colorado Beekeeper Mentorship Program Pilot

JonesKurt M.Colorado State University Extension, Salida, CO, USA

In 2006, the beekeeping industry was alerted to Colony Collapse Disorder, a mysterious disorder in managed hives where 30–90 percent losses were reported. Through reporting of these large colony losses in the popular media, the public became even more aware of the importance of pollinators in our food supply and ecosystems. A new generation of hobby and part-time professional beekeepers was born. The number of new beekeeping clubs and individual memberships in state beekeeping associations continues to grow nationally, and in the Upper Arkansas Valley in central Colorado. Chaffee County Extension Director Kurt Jones created an official volunteer program entitled the Colorado Beekeeper Mentorship Program with the aim to recruit and train a cadre of volunteers who could work with novice beekeepers and promote scientifically-based beekeeping and integrated hive management principles. Volunteers were selected based on an application and screening process which included reference checks, criminal and motor vehicle histories, interviews, and beekeeping experience. Upon admittance into the program, volunteers participated in a seven-session course. Researchers, extension specialists, extension agents and experienced beekeepers taught classes aimed at mentoring novice beekeepers. Subjects included starting the apiary, equipment needs, safe handling and establishment of bee hives, disease recognition and abatement, bee nutritional needs, high altitude plants, seasonal management needs, and the art of mentorship. The Colorado Beekeeper Mentorship Program was sponsored by CSU Extension and Western SARE with assistance from the Central Colorado Beekeepers Association. The 2016 program graduates reported outreach activities including coordination of local beekeeper association educational activities, and one-on-one hands-on mentoring of some local beekeepers. One of last year’s graduates serves on the local NRCS Conservation Board, and is involved in a project to create additional beekeeper habitat in conjunction with the local Farm to Table farm, which provides fresh produce for area food banks and kitchens. Creation of pollinator habitat in the fringes will help with soil stabilization and hopefully will provide honey as a staple to compliment the produce outreach. Another 2017 graduate has recently been elected president of the local beekeeping club, and is offering high quality educational programs for the general public and for club members. More than 115 people participated in beekeeping educational programs offered by CSU Extension in Chaffee County in 2016, and an additional 102 were trained in 2017. All of the participants have reported learning about sustainable hive practices. Demand is high to repeat the Colorado Beekeeper Mentorship Program in 2019 locally. Four additional counties have also expressed interest in implementing the Colorado Beekeeper Mentorship Program (El Paso, Adams, Archuleta and Mesa County), and eight extension agents have expressed interest in attending an in-service program in 2018/2019 should one be offered (planned for Salida and Fort Collins, CO). This presentation will cover the details of the Colorado Beekeeper Mentorship Program with emphasis on how others can replicate this program in their home counties.

### 2.3. Interactions among Body Size, Larval Rearing, and Adult Environment Affect Drone Reproductive Development and Age of First Flight

MetzBradTarpyDavidNorth Carolina State University, Raleigh, NC, USA

Drone quality is critical for queen mating health and longevity, and thus affects colony growth and development. Colonies produce thousands of drones that congregate in mating leks (drone congregation areas, or DCAs) and compete for access to mate with queens. Larger drones tend to be present in higher numbers at DCAs and are more successful at mating. Further, some colonies produce drones that, apart from body size, are more likely to successfully mate and whose genetics are more highly represented in the worker population than their relative sperm contributions would suggest. Nevertheless, smaller drones persist in the population, still gaining some share of mating success, may be cheaper to produce, and may exhibit differential life history traits leading to distinct mating strategies. Therefore, it is necessary to study the physiological trade-offs to reproduction among drones and particularly how drone traits vary to better understand how these trade-offs affect drone and colony fitness. In our study, we used a one-way entrance trap to capture drones as they attempted to leave their colony. We measured emergence characteristics of drones from several sources, which were then individually marked and placed into two different foster colonies to observe the interaction between rearing and adult environments on changes in body mass, reproductive development, and flight ontogeny. We found that colonies reared drones that were significantly different in body size, reproductive quality, and age of first flight. Adult rearing environment also had a significant impact on the age of first flight but not body size or reproductive quality. Finally, smaller drones were more likely to fly early in life, even though they are less fecund. Altogether, this points to an interaction among adult environment, larval environment, drone morphology, and reproductive traits that merits a deeper exploration into the parameters of reproductive success and how drone traits are passed to subsequent generations.

### 2.4. Migrating Giant Honey Bees Display Spatial and Temporal Fidelity to Thailand Stopover Site

RobinsonWillard S.Biology Department, Casper College, Casper, WY, USA

Populations of the giant honey bee, *Apis dorsata*, face many threats, but paramount among them is habitat loss. The forests of South and Southeast Asia, the habitat of the giant bee, face the most rapid deforestation occurring anywhere in the world. In fall, 2009 I discovered and reported on a site—a mango orchard along the Pai River in northwestern Thailand—where 16 colonies of migrating giant honey bees bivouacked in close proximity to one another. The following year 36 bivouacs gathered at the orchard in approximately the same time frame (Robinson, 2012 *PLoS ONE* 0044976). Apparently the colonies were resting before moving to higher altitudes and flowering plants as the dry season began. Informants have told me that giant honey bees use the same site at the same time every year; I tested that hypothesis by returning to the site in fall, 2016. That year, I counted 49 bivouacs (see Figure 1) in and near the orchard, spanning almost precisely the time period in which they appeared in 2010. 

The bivouacs also occupied almost exactly the same area as in 2010. The orchard affords remarkable opportunities to study bees as they migrate. Although some colonies perch high, observable only with binoculars, many rest within ~1–5 m, easily accessible, e.g., I was able to describe and document their migratory dances in preparation for departure flights, plus foraging, ventilation and defensive behavior. An excellent location for continuing research, the stopover site is probably not unique in southern Asia. Most likely such sites exist wherever giant honey bees undertake long seasonal migrations. I describe attributes of the site, e.g., abundant food and water availability, its location along a major river, and other possible navigational cues. I recommend researchers search for bivouacking sites, particularly along rivers, wherever giant honey bees migrate. Stopover sites are doubtless essential to the life history and health of migratory populations, and are thus deserving of conservation policies.

### 2.5. Investigating Colony-Level Health Traits in Canada’s Honey Bee Population

PernalSteve F.BorbaRenataHooverShelley E.CurrieRobert W.GuarnaMarta M.ZayedAmroFosterLeonard J.Agriculture and Agri-Food Canada, Beaverlodge, AB, Canada

The main causes of colony death, as reported by Canadian beekeepers, include high pathogen/parasite infestation levels (e.g., Varroa mites, *Nosema* spp.), poor quality queens and severe weather conditions. Every year, Canadian beekeepers purchase approximately 300,000 foreign queens and package bees to replace dead colonies or expand their operations. Such large-scale importation of stock has the potential to introduce undesirable pathogens or genetics and supply bees that have not been selected to survive in northern temperate climates. The overall goal of our ongoing research is to develop genomic and proteomic markers for 12 economically-valuable traits (colony phenotypes), which will enable local queen producers to rapidly select and breed healthy and productive colonies that are well adapted Canadian to conditions. Based on the rich dataset resulting from colony evaluations from this project to date, this analysis will focus on the relationships among colony phenotypes and the dynamics of diseases and parasites on colony health and productivity. We are also evaluating the relative importance of these factors on winter mortality.

In the first year of our study, 1025 colonies from across Canada were phenotyped for the following colony-level traits: Varroa mite population growth, grooming behavior, hygienic behavior, defensiveness, honey production, brood area, pathogen abundance, innate immunity, gut microbiota and overwintering success. As anticipated, significant correlations were found among similar productivity phenotypes such as fall and spring colony weights (*r*^2^ = 0.8374; *p* < 0.001), as well as between instantaneous and total honey production (*r*^2^ = 0.6905; *p* < 0.001). We also determined that decreased hygienic behavior was associated with higher Varroa mite population growth rates and greater DWV-A copy numbers. Increased colony mite populations were also associated with lowered mite resistance and higher levels of DWV-A and DWV-B. We further determined that increases in DWV-A and decreases colony weight were highly predictive of winter mortality.

We are continuing to model and analyze these data with the goal of disentangling the inter-correlation of several important pathogens and phenotypes, so as to better understand drivers of the associations between pathogens, colony health and productivity. Progress in identifying proteomic and SNP markers for economically-desirable traits is ongoing, which we feel will enable enhanced methods for trait selection.

### 2.6. Working with Mite-Biter Bees: Recent Extension Efforts and Stock Evaluations

HuntGreg J.GivenKrispnEvansMatthewShennefieldDavidPurdue University, West Lafayette, IN, USA

Beekeepers often experience high colony losses associated with *Varroa* mites and virus when purchasing queens from out-of-state. A USDA project involving beekeepers in seven Midwestern states was initiated to promote the use of locally produced queens and mite resistant stocks, especially the “mite-biter” bees that have been selected for increased grooming behavior. Beekeeper collaborators have conducted numerous queen rearing courses and workshops on how to use local queens and nucs to promote a sustainable solution to our *Varroa* mite problem. An important aspect of the beekeeper extension efforts was the formation of new state queen breeding associations and websites that help to connect queen producers with beekeepers that want local queens. A previous stock comparison between Carniolans and mite-biters showed that the mite-biter queen colonies had one third the level of mites and about 2.5-fold higher survival than commercial-source queens. Our most recent comparison involved just one grafting source of queens selected for high mite-biting, and one source of Italian queens from a western supplier. The mite biter queen colonies again had about one third as many mites in the fall and much higher survival towards the end of winter. Although selecting for this trait (the proportion of chewed mites on sticky sample sheets) is problematic and labor intensive, beekeepers are beginning to value this trait and to perform these measurements to improve mite resistance.

### 2.7. A Novel Assay for Measuring Honey Bee Hygiene, and Predicting Colony-Level Varroa Resistance

WagonerKairaBelloJanMillarJocelynSpivakMarlaSchalCobyRueppellOlavDepartment of Entomology, University of North Carolina, Greensboro, NC, USA

Despite evidence of reduced pathogen loads in honey bee stocks selected for hygienic behavior, reports of the efficacy of hygienic stocks against the parasitic mite *Varroa destructor* (Varroa) are variable, and Varroa remains a critical threat to honey bee health. In an effort to enhance hygiene selection tools, we identified chemicals present in higher amounts on the cuticle of Varroa-infested and Deformed Wing Virus infected honey bee brood, as compared to healthy brood. These and similar chemicals previously associated with hygienic behavior were synthesized and used to develop and validate a bioassay for triggering hygienic uncapping and removal behavior. Results from bioassay trials in thirteen colonies from five different breeding backgrounds suggest that colony response to the chemical cues is a better predictor of colony mite load and mite removal efficiency than is response to the liquid nitrogen freeze-killed brood assay. In addition, the new chemical cue assay is faster, easier to use, and more biologically relevant than existing alternatives. This assay may serve as an improved tool for breeders and beekeepers interested in measuring honey bee hygiene levels for selection and/or management purposes. Adoption of this assay has the potential to lead to healthier honey bees, with greater resistance to Varroa and its associated pathogens.

### 2.8. A Longitudinal Study of the Principle Factors Leading to Colony Losses in Migratory Beekeeping

O’Shea-WhellerThomasSimone-FinstromMichaelDankaBobRinkevichFrankHealyKristenPennHannahSwaleDanielLangSarahFellowsC.J.Department of Entomology, Louisiana State University, Baton Rouge, LA, USA

Commercial beekeeping in the United States accounts for ~3/4 of all colonies in circulation, with migratory pollination comprising a large proportion of the industry. However, the system simultaneously experiences high overwinter losses on a consistent and yearly basis. Consequently, without substantial improvements in colony health, large-scale migratory pollination is likely to become both biologically unsustainable, and commercially infeasible in its current form. Our project aims to identify the key predictors of colony loss and their relative importance, both at a large spatiotemporal scale, and under real-world conditions (Figure 2). Additionally, our study incorporates a field test of Pol-Line bees, a stock developed for resistance to Varroa mites, which current data implicate as the principle extraneous threat to bee colonies in the United States. Our results demonstrate the relative weightings of parasite, disease, forage and pesticide stressors upon colony health. Crucially, we show that Varroa accounts for ~70% of observed mortality across regions. Furthermore, Pol-Line bees were found to experience a marked and significant reduction in mortality, and consistently lower mite levels throughout the year, presenting a viable alternative to current commercial stocks. In sum, our data suggest that Varroa should be the prime concern if colony losses are to be curtailed, and that integrated control methods, principally in the form of mite resistant stocks, are the most effective long-term solution to the current *V. destructor* pandemic.

### 2.9. Interactions between Environmental Stressors and Honey Bee Immunity

O’NealScott T.AndersonTroy D.Department of Entomology, University of Nebraska, Lincoln, NE, USA

The honey bee (*Apis mellifera*) plays an economically vital role in global agriculture as a pollinator of a wide variety of crops, in addition to being valued for the honey and other natural products that it provides. Declines in the numbers of both managed and wild pollinators have increased public awareness of bee health issues and prompted researchers to intensify efforts to understand the forces driving these declines. While there exist a variety of factors that negatively impact the health and survival of both managed and wild bee populations, there is a growing consensus that high levels of parasites and pathogens, especially viruses, are among the most significant threats to the health of managed bees. Unfortunately, one of the longstanding challenges associated with bee research is the lack of tools and standardized approaches for the study of host/pathogen interactions in bees, in particular interactions between viruses and the antiviral immune response of bees. This presentation will examine how the study of bee immunity is evolving and explore some of the recent advances that will assist in understanding the complicated network of interactions between stressors that negatively impact the health and survival of managed bees.

### 2.10. Optimizing the Benefits of Propolis to Honey Bee Health in Beekeeping Operations

ShanahanMaggieSimone-FinstromMichaelSpivakMarlaDepartment of Entomology, University of Minnesota, St. Paul, MN, USA

Wild honey bee colonies coat the rough inner surfaces of hollow tree cavities with propolis, a substance comprised primarily of plant resins, and the resulting “propolis envelope” serves both structural and therapeutic functions inside the hive. Though previous studies have shown that the presence of a propolis envelope leads to both individual and colony-level health benefits through the modulation of immune gene expression and increased colony strength, propolis has yet to be implemented as a tool to boost colony health in day-to-day beekeeping operations. Moreover, the smooth surfaces of the standardized wooden bee boxes currently used in beekeeping do not encourage bees to build a propolis envelope (Figure 3). In this study, we examined how to best stimulate colonies to construct a propolis envelope that provides significant health benefits. Five different treatment groups were tested: (1) a control group using standard smooth bee boxes without propolis traps, (2) unfinished bee boxes with rough interior surfaces, (3) bee boxes with a “striated” or “ridged” texture, (4) conventional propolis traps placed on three interior hive walls and entrance, and (5) conventional propolis traps placed on a single interior hive wall and entrance. Standard indicators of colony health were measured, and bee samples were collected to test immune gene expression using quantitative PCR techniques. The results of this study will help beekeepers optimize the benefits of the propolis envelope in their operations.

### 2.11. Nosema Ceranae and Immune Inducers: Their Effects on Honey Bee Longevity and Behavior

Guzman-NovoaErnestoValizadehPegahGoodwinPaul H.Correa-BenitezAdrianaUniversity of Guelph, Guelph, ON, Canada

*Nosema ceranae*, the dominant microsporidium causing nosema disease of honey bees (*Apis mellifera*) worldwide has been linked to cases of colony losses. The antibiotic fumagillin has been used for 60 years to control this disease, but its production has been discontinued or its use banned in many countries. Beekeepers thus, need alternative treatments against nosema disease. Several microbial-derived compounds were tested for their ability to induce an immune response against *N. ceranae* and to inhibit its reproduction as an alternative treatment to fumagillin. Two selected compounds, chitosan and peptidoglycan, were also studied for their effects on the survivorship, hygienic behavior and foraging behavior of honey bees infected with *N. ceranae*. Bees inoculated with the parasite were treated with these compounds and kept in incubators and observation hives. Chitosan and peptidoglycan significantly restrained the development of *N. ceranae* infections and altered the expression of immune-related genes, such as hymenoptaecin and defensin 2. *N. ceranae* infections reduced the survivorship of bees, whereas chitosan and peptidoglycan significantly lowered the mortality caused by the parasite. Neither *N. ceranae* infections nor the compounds tested, altered the expression of hygienic behavior of worker bees. However, both compounds significantly increased the frequency of foraging trips in treated bees, compared to *N. ceranae* infected and non-infected bees. The results of this study support the hypothesis that chitosan and peptidoglycan have beneficial effects on honey bee health and foraging behavior in addition to their potential effects on restraining *N. ceranae* infections. Therefore, chitosan and peptidoglycan warrant testing in field colonies.

### 2.12. Gluconic Acid for the Control of Nosema Ceranae

VincentJacobWebsterThomasKammingaKatherineKentucky State University, Frankfort, KY, USA

One cause of recent honey bee colony decline is the microsporidian *Nosema ceranae*. In the U.S., this pathogen is now more prevalent than *N. apis* in *Apis mellifera*. Fumagillin has been a common treatment for *N. apis*, but studies on its efficacy for *N. ceranae* control are contradictory. Furthermore, fumagillin is no longer sold for this purpose in the U.S. Earlier work at Kentucky State University showed that honey inhibits the development of *N. ceranae* in honey bee midguts. Possibly this is due to organic acids, thought to contribute to honey’s antibacterial and antifungal qualities. Gluconic acid is the most abundant organic acid in honey constituting about 0.5% of honey by weight. Due to the need for alternative treatments for *N. ceranae*, we tested the ability of gluconic acid to inhibit the growth of *N. ceranae*. Caged honey bees were inoculated with 20,000 *N. ceranae* spores per bee in a sugar syrup solution. The bees were then randomly separated into a control and treatment group, and placed into an incubator set at 32 °C. The control group was fed a 60% sucrose solution ad libitum and the treatment group was fed a 60% sucrose solution containing 0.5% gluconic acid. 10 days post-inoculation, 8 midguts were collected from bees from both groups, macerated, and a spore count was acquired using a hemacytometer. The control group had a spore count of 7.6 million while the treatment group had no visible spores. These preliminary results indicate that gluconic acid could be a viable treatment for *N. ceranae* infection.

### 2.13. Nosema Ceranae Causes Peritrophic Matrix Diassociation from Midgut Tissue in Worker Honey Bees

WebsterThomas C.KammingaKatherine L.Kentucky State University, Frankfort, KY, USA

A longitudinal study of *Nosema ceranae* infection in worker honey bees was conducted in order to better evaluate the natural progress of the disease, and how therapeutic agents might function most effectively. We have a particular interest in the peritrophic matrix (PM) of the bee, because it plays several significant roles. The PM inhibits invasion by pathogens, acts as a substrate for enzymes important to food digestion, and moves food through the midgut in a timely manner. Worker bees were infected individually, by feeding each with 20,000 *N. ceranae* spores in 50% sucrose syrup. The bees were then kept caged in an incubator at 32 C for 12 days, during which time they were fed 50% sucrose and water in separate feeders, ad libitum. Beginning four days after inoculation, five bees were removed from their cages every two days. Midguts were removed from these bees, fixed in formalin and then (a) passed through a standard series of dehydration steps using ethanol and xylene substitute, (b) embedded in paraffin, (c) sliced so that 5 micron sections were laid onto microscope slides, (d) re-hydrated, and (e) stained with Biebrich and calcofluor white. Examination by fluorescence microscopy showed midgut tissue, including the PM, and *N. ceranae* spores inside of the midgut host cells. At four and six days post-inoculation (dpi) the PM adhered closely to the midgut epithelial cells, as in healthy bees. At eight, ten and twelve dpi the PM was no longer attached to the epithelial cells, and no PM secretion could be observed. These observations support the hypothesis that *N. ceranae* inhibits the secretion and function of the PM. Examination of the entire midgut showed that infection was confined primarily to the posterior third of the midgut.

### 2.14. European Foulbrood and Fungicides in Honey Bees during Blueberry Pollination

MilbrathMeghan O.GrahamKelsey K.IsaacsRufusMichigan State University, East Lansing, MI, USA; mpi@msu.edu

Michigan is one of the top blueberry producing states in the country, and Michigan growers depend heavily on commercial honey bee pollination services. Commercial beekeepers often report that their colonies suffer from high rates of European Foulbrood Disease (EFB) after completing blueberry pollination contracts. EFB is a bacterial disease of honey bees that is linked to poor health outcomes as well as loss of honey production. We investigated the relationship between EFB and blueberry pollination by following the health outcomes of colonies in 14 different blueberry fields. We hypothesized that EFB could be related to two stressors: protein deficiency and fungicide exposure. To identify if protein deficiency was a factor, we performed a simple cohort study to identify if pollen exclusion or protein supplementation affected incidence of EFB. To identify if fungicide exposure affected rates of EFB, we measured differences in EFB incidence in fields that were conventionally sprayed compared fields that were not sprayed during that season, and compared levels of incoming fungicides. Pollen was collected continuously through the study period to identify food sources as well as levels of fungicides exposure. Finally, we followed a subset of the infected colonies to identify rates of recovery in relation to antibiotic use and pollen supplementation. We found very high rates of European Foulbrood after blueberry pollination, indicating that this remains a serious concern for beekeepers.

### 2.15. Vertical Virus Transmission in Honey Bees: What Is in the Egg?

AmiriEsmaeilHermanJacobStrandMicheline K.TarpyDavid R.RueppellOlavDepartment of Biology, University of North Carolina, Greensboro, NC, USA

Honey bees are social insects that live in colonies, where their survival and fitness are inextricably linked to specialized reproductive individuals (queens) that monopolize the egg laying. The queen is the longest-lived individual of the colony, and as such is exposed to many different stresses, including viruses. As the sole egg layer, queens also transmit viruses widely to the next generation. Virus infection can also elicit transcriptional responses in queens and her eggs to potentially lower offspring susceptibility to the virus. To understand the dynamics of vertical transmission of honey bee viruses, we investigated the virus transmission through eggs. We surveyed the virus titers of pooled eggs from 85 queens heading commercial colonies, and we investigated the transcriptional profile of virus-infected and non-infected eggs via RNA sequencing. Together with our previous results, these studies allow us to assess associations between queen health status, transgenerational virus communities, and transgenerational immunity. Our results are discussed in the context of the central role of the queen for honey bee health.

### 2.16. Investigating the Impact of Honey Bee Genotype and Varroa Destructor Mites on Virus Prevalence and Abundance

ParekhFenaliFaurot-DanielsCayleyDaughenbaughKatie F.GivenKrispnHuntGreg J.FlennikenMichelleMicrobiology and Immunology, Pollinator Health Center, Montana State University

Honey bees (*Apis mellifera*) are important producers of honey as well as play a vital role in pollination of numerous crops. Since 2006, average annual honey bee colony losses in the US have averaged approximately 33%. Colony losses are associated with several abiotic and biotic factors including *Varroa destructor* mite infestation and virus infection. To further investigate the impact of honey bee genotype and *Varroa destructor* mites on virus prevalence and abundance we obtained honey bee and mite samples from 36 colonies (i.e., 18 Purdue genotype colonies and 18 commercial colonies) and evaluated the prevalence of nine and abundance of three viruses. In addition, negative strand-specific PCR was utilized to determine if select viruses were actively replicating in honey bee and mite samples. The results from this small study indicate that deformed wing virus and black queen cell virus prevalence was similar in the two honey bee stocks (which were sampled on October 30, 2017). In general, deformed wing virus levels in bee and mite samples correlated and scaled together, whereas LSV1 levels were typically higher in bee samples compared to the corresponding mite samples. Many bee samples had moderate to high black queen cell virus (BQCV) levels, but very low to no BQCV was detected in corresponding mite samples. Together our data supports previous findings indicating that DWV replicates in *V. destructor* mites, as well as in honey bees.

### 2.17. Spillover of RNA Viruses from Managed Honey Bees to Wild Bumble Bees

AlgerSamantha AnnBurnhamP. AlexanderBoncristianiHumberto F.BrodyAlison K.University of Vermont, Burlington, VT, USA

RNA viruses, once considered specific to honey bees, are suspected of spilling over from managed honey bees into wild bumble bee populations. To test this, we collected bees and flowers in the field from areas with and without honey bee apiaries nearby. Prevalence of deformed wing virus (DWV) and black queen cell virus (BQCV) as well as replicating DWV infections in *Bombus vagans* and *B. bimaculatus* were highest in bumble bees collected near honey bee apiaries (χ 12 < 6.531, *p* < 0.05). Our results suggest that honey bees are significant contributors of viruses to bumble bees. Flowers have been suspected as bridges in virus transmission among bees. We detected bee viruses on 18% of the flowers collected within honey bee apiaries and detected no virus on flowers in areas without apiaries, thus providing evidence that viruses are transmitted at flowers from infected honey bees. In controlled experiments using captive colonies in flight cages, we found that honey bees leave viruses on flowers but not equally across plant species. My results suggest that there are differences in virus ecology mediated by floral morphology and/or pollinator behavior.

### 2.18. Spillover of RNA Viruses between Apis mellifera and Bombus spp. through Shared Flowers Diluted by Floral Diversity

BurnhamP. AlexanderAlgerSamantha A.BoncristianiHumbertoVermont Complex Systems Center, University of Vermont, Burlington, VT, USA; alexburn17@gmail.com

Evidence is mounting that RNA viruses, likely originating from honeybees, have been spilling over into wild bee populations. While transmission of disease between bee species likely occurs through the use of shared flowers, only two published studies have directly examined this floral transmission route and no study has yet examined their role in RNA virus dissemination. Previous work in this system has shown that flowers can harbor viruses and that prevalence varies across species. In the field of disease ecology, the dilution hypothesis examines the idea that host diversity can dampen the effectiveness of a pathogen reducing the prevalence of the disease. Though there is evidence to support this hypothesis, the underlying mechanisms are still undetermined and the debate over its existence persists. In this study, we experimentally demonstrate how flowers may be facilitating the spillover of Deformed Wing Virus (DWV) from honey bees into bumble bee communities. In addition, through a combination of experimentation and mathematical modeling, we propose a mechanism for the dilution of transmission through increased floral diversity. Clean bumble bees were allowed to forage on hand inoculated and naturally infected artificial and natural flowers. Viral load was measured using RT-qPCR. We measured the viral load that can be picked up by a bee from a flower as a function of foraging time, determined the potential rate at which the route might work in reverse (bumble bees to honeybees) and determined how much virus is required to cause a replicating infection. We found that DWV can be picked up by bumble bees from field realistic hand-inoculated flowers and that foraging time increased viral load only during the first 10 s of visitation. Our mathematical model showed that the parameter estimates for viral deposition and probability of infection are able to drive a simulated spillover event that mirrors empirical data from field surveys. The inclusion of floral diversity with differential viral harboring potential dampened overall disease prevalence. Spillover of diseases into naïve bumble bee populations has been implicated as one of the factors responsible for their losses. The demonstration of this transmission route and the proposed floral dilution mechanism may be used to inform recommendations for responding to future emerging infectious disease outbreaks. Future work should look to optimized tolerance (HOT) models, which might be used to determine ideal floral composition for human-made pollinator gardens and crop buffers to maximize floral diversity and minimize disease spillover risk.

### 2.19. Spillover in Eusocial Insects: Detection of Honey Bee (Apis mellifera) Associated Viruses in Ants

PayneAlexandria NRangelDr. JulianaDepartment of Entomology, Texas A&M University, College Station, TX, USA

Ants are often considered pests of honey bees (*Apis mellifera*) within managed apiaries. They have been known to: (1) rob nectar resources from weak or collapsed honey bee colonies, (2) scavenge off of dead bees and brood outside and within hives, and (3) live within honey bee hives. However, despite their common presence in or around honey bee colonies, we still do not know how ant species interact with honey bees when robbing from or living within their hives. This is especially true regarding any form of disease transmission that may be occurring between these two eusocial insects. Three studies done in France/New Zealand found the presence and active replication of honey bee-associated viruses in ants, but it is not yet known if this same phenomenon occurs in the United States. This study aims to screen different species of ants for six commonly found honey bee associated viruses: Deformed Wing Virus (DWV), Black Queen Cell Virus (BQCV), Israeli Acute Paralysis Virus (IAPV), Acute Bee Paralysis Virus (ABPV), Kashmir Bee Virus (KBV), and Sacbrood Bee Virus (SBV). Sampling occurred across the state of Texas at both apiary and non-apiary locations. We conducted diagnostic analyses using PCR to screen for viral presence and then conducted strand-specific RT-PCR to determine if active replication of our viruses of interest were occurring in ants both within and outside of apiaries. Preliminary research has demonstrated some species of ants are hosts of honey bee-associated viruses and should be considered when developing control strategies to curtail the spread of viruses between honey bee colonies.

### 2.20. Honeybee Behavioral Resilience to Varroa Mite Challenges

Li-ByarlayHongmeiSmithJadaAgricultural Research and Development Program, Central State University, Wilberforce, OH, USA

The honeybee is the most important managed pollinator for sustainable agriculture. However, American beekeepers experience 30–40% of managed colony losses since 2008. Among all the biotic stressors, the parasitic mite *Varroa destructor* is the top driver for the colony collapses. Feral colonies may be behavioral resilience to *Varroa* mites due to natural selection. Candidate feral bee colonies were tested in Central and Western Ohio for mite biting behavior. Our central hypothesis is that feral colonies display higher mite biting behavior than the commercial package colonies. Furthermore, the mandibles of worker bees from these two populations were also compared by micro-CT scanning. We have surveyed mite biting behavior in 39 colonies in 2018. Our results showed the first scientific evidence of the mite biting behavior in Ohio feral bee population and its potential defensive mechanism with mandibles. We provide a piece of significant knowledge in the breeding of mite resistant bees in Ohio and the Midwest.

### 2.21. Impact of Stressors on Grooming Behavior and Gene Expression of Honey Bees (Apis mellifera L.)

MorfinNuriaGoodwinPaul H.Guzman-NovoaErnestoUniversity of Guelph, Guelph, ON, Canada

Honey bees (*Apis mellifera* L.) are constantly exposed to biotic and abiotic stressors, like parasites and insecticides. It is known that honey bees perform grooming behavior to remove pathogens from their bodies as a defense mechanism. Although research has been done on the mechanisms that govern grooming behavior, little is known about the effect of biotic and abiotic stressors on grooming behavior and the possible impact on honey bee health. To evaluate the effect of biotic and abiotic stressors and their combined effects, honey bees were exposed to three different sublethal doses of the insecticide clothianidin and/or *Varroa destructor* for seven consecutive days. Through an evaluation of individual grooming behavior, this study found that sublethal doses of clothianidin, *V. destructor*, and their combination significantly reduced the proportion of bees that groomed intensively. Also, an interaction between the stressors was found in the expression of the gene neuroligin (*AmNlg-1*), which codes for a postsynaptic protein that interacts with the protein neurexin during synapse. Neurexin, coded by *AmNrx-1*, has been associated with grooming behavior in bees. Thus, these results suggest that the stressors, alone and combined, affect neural processes and the ability of the bees to remove pathogens from their bodies, which could have an impact on honey bee health and consequently contribute to colony mortality.

### 2.22. Controlled Time-Release of Formic and Oxalic Acids for Varroa Control

StarkEdmund J.MilbrathMeghan O.ReidNathanMcLeanTrentonLaurinJacquelinHenningRebeccaMackinKeegan I.Michigan State University, Midland, MI, USA

Since its introduction to the US in the late 1980s, the Varroa mite has been the most serious pest in the beekeeping industry and is a primary cause of honey bee colony loss. Over the last decade, beekeepers in the U.S have lost a third of their honey bee colonies each year. Mites have developed resistance to earlier synthetic acaricides, and may be developing resistance to Amitraz as well. All synthetic treatments have significant limitations, among which are: expensive, labor intensive, unsafe to apply, toxic to developing bees, queen loss, toxic in honey, or build-up in wax. A safe and effective natural treatment for the Varroa mite is desperately needed. Formic acid and oxalic acid are naturally derived miticides, and resistance to them has not been observed. Formic acid is labeled for use during honey production. Newly registered oxalic acid is not yet labeled for such use in the US, but is effective during the broodless period. Unfortunately, current oxalic acid treatments are “homemade,”resulting in variable doses, and can be hazardous to the applicator. Formic acid, as MAQS, effectively releases formic acid over several days, depending on hive conditions. MAQS cannot be used in much of the country during honey production, because outside temperatures are above those that are safe for useMSU has developed a controlled-release formulation for formic acid and oxalic acid that has the potential to be used as an effective miticide in honey bee hives. The strategy involves hyperbranched poly(esters) (HBPE) from “natural”, biodegradable building blocks to which the active ingredient is covalently bonded. As the HBPE degrades, it releases the miticide in a slow, controlled rate. The release rates of these formulations have been studied in numerous laboratory trials. The rate is dependent on temperature and humidity, but the release of formic acid from the HBPE is slower than MAQS under similar conditions, and lasts long enough to kill mites in the brood cycle, not just phoretic mites. The cost of the raw materials is low and the synthetic process is simple and inexpensive. Most importantly, initial hive tests were successful, and synthesis of the miticide has been scaled up to allow testing in 54 hives in 3 yards, including controls. Five different dose levels were tested, and two application methods. Dosages consistent with early work remained effective in this larger study, whereas lower doses lost effectiveness. Application using BeetleBlaster traps proved ineffective.

### 2.23. Varroa Mite Impacts on Queen Bee Quality in the Hawaiian Islands

RusertLauren M.TarpyDavid R.PettisJeff S.Department of Entomology & Plant Pathology, North Carolina State University, Raleigh, NC, USA

In the midst of widespread pollinator declines, including high annual losses of *Apis mellifera* (honey bees), beekeepers struggle to pin-point the exact cause of their loss. While many factors impacting *A. mellifera* declines have been studied, little is known about the impact of pre- and post-Varroa mite introduction on the mating frequency and quality of queen bees. Since the arrival of Varroa mites into the U.S. over 30 years ago, beekeepers have faced many setbacks to keep their hives healthy. Varroa has affected beekeeping worldwide, leaving only a few places untouched by this devastating pest, including several of the Hawaiian Islands. Hawaii is a unique place to study bees not only for the lack of Varroa, but also because of the strict regulations on inter-island bee movement as well as it being home to some of the largest queen breeders in the world. For my project, I mated queen bees on four Hawaiian Islands: Kauai, Oahu, Maui, and Hawaii island, and collected their offspring to look at queen mating frequency and mating quality between the islands. Using microsatellite DNA markers, worker bees are being analyzed for the number of paternal lines in each colony to see how genetic diversity is affected. We will see whether islands with Varroa mites or without have a greater impact on mating frequency and the quality of queens. This information will give us insight on whether Varroa mites actually have an impact on genetic diversity and whether there are other major factors contributing to colony decline. With the eventual introduction of Varroa mites to the rest of the world, time is limited to study the areas untouched by this devastating pest.

### 2.24. Phoretic Varroa Mite Reduces Flight Performance in Honey Bee Foragers

HuangZachary Y.CuiJianxinFuXiaoweiZhangZhongyinLiuHaoDepartment of Entomology, Michigan State University, East Lansing, MI, USA

*Varroa destructor* has been shown to affect honey bees negatives in many areas: decreased body weight, shortened lifespan, lowered foraging efficiency, and reduced mating ability in drones. Mite infestation during pupal stage reduced flight time in workers, but whether mite infection during the adult stage also affected flight ability was not studied. We inoculated returning foragers with 1 or 2 mites and compared to a control group (0 mite per bee) and measured their flight ability on flight mills. We found a significant effect of varroa mite on the following parameters: total flight distance, total flight duration, number of flight bouts, flight distance in the longest flight bout, and flight duration in the longest flight bout. Flight speed and flight speed in the longest flight bout were not affected. Our data suggest that varroa on adult workers either reduce flight ability by affecting aerodynamics (unlikely) or via feeding damage during the phoretic stage (more likely).

### 2.25. When Would a Honey Bee Advertise Flowers in a Restored Prairie and Why Does It Matter?

Carr-MarkellMorgan K.DemlerCora M.CouvillonMargaret J.SchürchRogerCornmanR. ScottIwanowiczDeborah D.SpivakMarlaDepartment of Entomology, University of Minnesota, St. Paul, MN, USA

Abundant nectar and diverse pollens help honey bee colonies fight off diseases and detoxify pesticides. Unfortunately, recent increases in corn and soybeans in the Upper Midwest region have decreased the food sources available to bees. Many groups are interested in planting native prairie flowers with the goals of restoring prairie ecosystems and helping honey bees. However, in designing these bee-friendly plantings, we must consider the foraging preferences and strategies of honey bee colonies. This leads to the question: when given access to restored prairies, what proportion of their diet will honey bee colonies collect from prairie flowers? To answer this question, we placed colonies near four large, restored prairies and examined the pollen coming into those colonies throughout the foraging season. We found little contribution from native prairie species until the late summer and fall, when native aster species, such as *Solidago* (goldenrods), became a major source of food. In the summer, our colonies collected large proportions of their pollen from three non-native genera: *Trifolium* (white/alsike/red clover), *Melilotus* (sweet clover), and *Lotus* (birds-foot trefoil). The honey bee foraging strategy involves communications among foragers about the locations of the most profitable patches of flowers. Therefore, to understand colony foraging decisions we asked: to what extent do honey bee foragers advertise flower patches in prairies? To determine more precisely where our bees found their preferred flowers, we placed glass-walled observation hives near two large prairies and decoded/mapped waggle dance communications between foragers in those hives. Maps of the dance decoding results showed that most flower patches advertised by foragers were outside of the restored prairies, but at one site we saw increasing use of prairie flower patches in the late summer and early fall. These seasonal patterns raise the question: what makes prairies more attractive at certain times of year? How do factors such as species, floral density, and floral diversity affect honey bees’ evaluations of flowers in prairies and thus colony-level foraging behavior? We are now analyzing data from an experiment testing the effects of flower density on honey bee recruitment. Future studies could also test the effects of species composition and floral diversity and help us determine how to design native plantings to maximize their attractiveness to honey bee colonies.

### 2.26. Bioindicators for a Sustainable Future: Dancing Honey Bees Communicate Habitats’ Ability to Feed Pollinators

CouvillonMargaret J.Virginia Polytechnic Institute, Blacksburg, VA, USA

Lack of forage is a factor contributing to bee declines. This stressor can act directly, where hungry bees are unable to meet their nutritional needs, or indirectly, where the resulting nutritional stress reduces the bees’ ability to cope with other stressors like diseases and pesticides. Coverage has been wide: everyone wants to feed hungry bees. Such help is offered with best intentions, but efficacy is undermined by two crucial knowledge gaps: firstly, we do not know when and where bees lack forage. Providing flowers indiscriminately is a common practice because current methods of surveying, cataloging, and comparing floral abundance at a landscape-scale is intensely time-consuming. Secondly, nutritional stress is often studied either in honey bees (*Apis mellifera*) or non-honey bees, creating a dichotomy that limits the usefulness of resulting recommendations. For example, we do not know how *Apis* forage relates to habitat quality for other pollinators. Thus, there is a critical need to develop new methods to survey *Apis* forage on a landscape scale and to determine if landscapes preferred by honey bees are additionally preferred by non-*Apis* bees.

In my lab, we are using honey bees in three distinct environments in Virginia to generate biologically-relevant data on forage availability primarily, but also pesticide exposure. We will use methods that we piloted with honey bee waggle dances, a form of communication where successful foragers indicate to nestmates the vector to food. Waggle dances are visible to the eye and can be decoded and mapped. Thus, we can determine both when and where bees are collecting food in a landscape. Importantly, we will then conduct experiments to determine if our results are pertinent to a range of pollinating bees. Specifically, we hypothesize that honey bee data on forage availability will positively correlate with diversity, abundance, and survival of wild and bumble bee colonies. Our research has two long-term goals. First, we will contribute to best management strategies to improve pollinator nutrition by generating data on when and where supplemental forage is most needed. Second, we will determine if honey bees can act as bioindicators to inform on a landscape’s ability to feed pollinators. These data will help us implement a best management strategy for improving availability of nutritious forage that would be beneficial to overall pollinator health in a meaningful, targeted way.

### 2.27. Establishing a Correlation between Soybean Presence and Soybean Foraging by A. mellifera

HearonLuke E.JohnsonReed M.LinChia-HuaThe Ohio State University, Columbus, OH, USA

Soybean farming contributes a large portion of Ohio’s annual crop revenue and accounts for a similarly large portion of Ohio’s cropland. Though soybean (*Glycine max*) is self-pollinated, and thus not dependent on insect pollinators, it has been found that insect pollinators will visit soybean flowers nonetheless, and in doing so cause an increase in soybean yield. Conventionally, soybean has not been considered a major source of nectar for the summertime foraging of honey bees in Ohio. Instead, white clover (*Trifolium repens*) is often cited as the primary nectar source. We seek to determine the extent to which *G. max* contributes to honey bee nectar collection. We examined pollen extracted from 40 honey samples from across the state of Ohio using light microscopy. We counted the number of soybean pollen grains, the number of clover pollen grains, and total number of pollen grains. We then usee geographic information system (GIS) analysis to determine the area of soybean cropland within foraging range of the hives at the time samples were taken. We will compare the soybean:clover ratio to the area of soybean in the landscape and correlated the two. This provides a proxy of honey bee preference for *G. max* given the availability of this floral resource around each apiary. We predicted that as area of *G. max* increased in the surrounding landscape, the ratio of *G. max* pollen count relative to *T. repens* in honey samples would increase. The implications of heavy foraging on *G. max* by honey bees are twofold; to the soybean grower and to the beekeeper. A clearer understanding of soybean pollination would allow farmers to make more informed choices about how and when to apply pesticides and beekeepers could position their hives to maximize productivity.

### 2.28. Everything’s Sweeter in Texas? A Chemical and Palynological Analysis of Honey in Texas

LauPierreBryantVaughnDübeckeArneRangelJulianaTexas A&M University, College Station, TX, USA

Honey bees (*Apis mellifera*) use nectar as their main source of energy to fuel colony growth and development. Nectar is converted to honey, which is increasing in demand for their health benefits in humans. Understanding the floral resources collected by colonies in different regions will aid in the promotion of plant species to enhance honey bee colony health and honey production. The significance of this research is two-fold. First, this study will help us identify and promote important plant species that honey bees forage on. This study will also help local beekeepers by establishing a baseline for detecting adulterated honey, as honey is commonly mixed with foreign products to improve economic yield. We conducted a palynological analysis of 119 honey samples provided by participating beekeepers whose colonies were located throughout Texas. The pollen in each honey sample was extracted, acetolyzed, identified, and classified in frequency categories depending on each taxon’s relative abundance. The water content, pH, and sugar spectrum were also analyzed with Nuclear Magnetic Resonance (NMR) spectroscopy. We have found at least 121 unique pollen types in honey including *Triadaca*, *Mimosa*, *Ulmus*, *Prosopis*, and *Rhus* (Figure 4). The water content in honey ranged from 14% to 20.6%, the pH ranged from 3.6 to 5.4, and the combined fructose and glucose content ranged from 50.7% to 77%. This information will help us better understand honey bee nectar foraging preferences in their environment. This will also serve as a foundation for future studies on promoting pure, unadulterated honey.

### 2.29. Dismantling Babel: Creation of a Universal Calibration for Honey Bee Waggle Dance Decoding

SchürchRogerDepartment of Entomology, Virginia Polytechnic Institute, Blacksburg, VA, USA

When a honey bee forager has found a good resource, usually the nectar or pollen that is her food, she recruits her nestmates by communicating the location of the forage with the waggle dance. This unique behavior therefore conveys biologically relevant information about where bees go to collect food. In a waggle dance, direction is encoded in the angle of the dancing forager’s body relative to up, and distance is encoded in the duration of the dance. In the past, researchers translated duration to distance by using the published calibration of Karl von Frisch; however, it has been suggested that landscape and subspecies might alter calibrations. Here we analyzed the waggle dances from individually marked honey bees (*n* = 859 dances from 85 bees from three hives) that foraged at multiple known locations to determine a distance-to-duration calibration. Next we compared this calibration to a previously published calibration, which demonstrated that while their slopes are similar (*p* = 0.82), their intercepts differ (*p* < 0.001); however, the individual variation, or noise, between bees is so high that this difference is rendered biologically irrelevant. We then collated our data with all published calibration studies to generate a Universal Calibration that incorporates the inter-individual and inter-study differences. Lastly, we verified that the Universal Calibration performs as well as a landscape/subspecies-specific calibration first with a hold-out sample of waggle dances (*n* = 84) (performance comparison, *p* = 0.36) and second with a linear discriminant analysis, which failed to assign dances to their originating population. Both these confirm that the Universal Calibration may be used by other researchers, irrespective of landscape or subspecies, to recover foraging locations from honey bee waggle dances. By allowing noise to be part of our analysis, the usability of our calibration is broadened for use in future settings.

### 2.30. Dancing Honey Bees Communicate Honey Bee Foraging Preferences in an Orchard and Food Crop Landscape

SteeleTaylor N.SchürchRogerCouvillonMargaret J.Virginia Polytechnic Institute, Blacksburg, VA, USA; taylorsteele@vt.edu

Honey bees (*Apis mellifera*) are important pollinators of crops such as alfalfa, apples, nuts, cantaloupe, cranberry, pumpkin, sunflower and vegetables. The value of honey bee pollination is worth more than seventeen billion annually in the United States. It has been widely publicized that pollinators have been declining. This decline has been associated with many interconnected factors including habitat loss and fragmentation, pesticide usage, disease, pests, and lack of forage. Lack of forage is one of the stressors that can directly affect the bees, causing malnutrition, but can also indirectly affect them, when malnutrition causes a reduction in their ability to cope with other stressors, such as disease, pests and pesticides. Currently, researchers do not fully understand the dynamics of food collection. In particular, we do not have a comprehensive knowledge of the foraging dynamics in orchard and fruit crop systems, where there might be a feast or famine situation. Lack of available seasonal forage in these systems may be affecting the health of honey bees and the landscape that supports them. To understand orchard and fruit crop systems usage by honey bees, we investigated foraging behavior at the Alson H. Smith Agricultural Research and Extension Center (AREC) in Northern Virginia. We decoded honey bee waggle dances from returning foragers communicating to nestmates a distance and direction, from the hive to the source of nectar, pollen, or other resources. Waggle dances are visible to the naked eye and able to be analyzed through video recording and spatially mapped to determine where bees are and are not foraging in this landscape. Decoding these dances will determine the contribution of fruit orchards to colony forage and, conversely, help estimate the contribution of honey bees to fruit orchard pollination in an effort to determine best management practices for improving food availability to benefit overall pollinator health.

### 2.31. Spatiotemporally Decoupled Effects of Land Use on Ecosystem Service Delivery

SmartMatthewOttoClintDepartment of Entomology, University of Nebraska, Lincoln, NE, USAU.S. Geological Survey, Northern Prairie Wildlife Research Center, Jamestown, ND, USA

Land use change, habitat loss and lack of forage have been implicated as factors involved in the decline of managed pollinators. Changes in land use in the Northern Great Plains (NGP) region of the US have resulted in shifts away from historical grassland ecosystems toward landscapes dominated by crops such as corn and soybeans. We discuss how land use during the growing season in the NGP impacts colony population size for almond pollination in central California in February. We estimate the magnitude of the effects of land use on the economics of beekeeping by linking summer habitat with pollination service payments and later production of new colonies. Our results demonstrate a greater amount of non-forage crops surrounding apiaries in summer results in colonies comprised of smaller population sizes during almond pollination. This latent effect of growing season land use impacts beekeepers with a reduced rental fee per colony for pollination services, and reduced potential for creating additional spring colonies via splitting. Overall, we highlight the downstream effects of land use, and factors driving land use decisions, on the ability of beekeepers to provide robust colonies to support the national pollination industry. We also demonstrate the relationship between habitat in the NGP, colony health, and pollination services provided elsewhere in the US.

### 2.32. Sublethal Effects of Neonicotinoid Pesticides on Honey Bee Foraging and Recruitment Behavior

OhlingerBradley D.SchürchRogerDurziSharif A.KietzmanParryCouvillonMargaret J.Department of Entomology, Virginia Polytechnic Institute and State University, Blacksburg, VA, USA

Foraging bees use their perception of reward to inform foraging decisions such as determining the food sources to exploit and the amount of effort to invest in individual foraging and colony-level recruitment. The ability of bees to make foraging decisions according to both the quality of the food sources and the needs of the colony is important to their foraging success and ability to provide valuable pollination services. However, not much is known about how neonicotinoid pesticides affect individual foraging and colony-level recruitment of freely flying bees in a field context. Recent studies report that bees cannot taste imidacloprid, but prefer to eat sucrose solutions laced with low but field-realistic levels of imidacloprid. These results suggest that foraging bees—unable to control their exposure to neonicotinoids—might experience enhanced reward perception and modified foraging decisions. In other words, neonicotinoids might be drugging bees, creating a positive feedback loop in which the drugged bees then increase their exposure. Given the widespread use of neonicotinoids and public concern surrounding these pesticides, research into the effects of these pesticides on honey bee behavior may provide much needed insight into the sublethal effects of pesticides on honey bees. We conducted a feeder experiment using freely flying bees to test for the effect of neonicotinoids on both individual foraging and colony-level recruitment in honey bees. In particular, we measured the effect of 100 nM imidacloprid (in 1 M sucrose solution) on foraging frequency, foraging persistency, site specificity, dance frequency, dance duration, and dance propensity compared to bees feeding on an adjacent equi-distant control feeder of 1 M sucrose solution. Foraging bees did not differ in the frequency of visits made to the treatment and control feeders. However, treated bees were 1.5 times more likely to drink from the alternate feeder than the control bees. Taken together, the results indicate that exposure to low concentrations of imidacloprid might not have a strong enough of a pharmacological effect on reward perception to alter individual foraging frequency in a field experiment, but might instead, contrary to previous research, elicit avoidance behavior by foraging bees.

### 2.33. Chlorothalonil Fungicide Effects on Honey Bee Hemocyte Respiration In Vitro

GoblirschMikeDusekCullenMartinovic-WeigeltDalmaDepartment of Entomology, University of Minnesota St. Paul, MN, USA

Chlorothalonil (2,4,5,6-tetrachloroisophthalonitrile) is a widely-used, broad-spectrum fungicide commonly applied to crops pollinated by honey bees. Its residues are frequently detected in wax and pollen sampled from the hive, and it has been detected in honey bee tissues (max concentration—870 ppb). This study evaluated the potential of chlorothalonil to affect cellular respiration of honey bee hemocytes in vitro. Hemocytes were collected in hemolymph after puncture of the dorsal vessel of fifth instar larvae. Hemocytes from individual larvae were seeded into wells of a 24-well microplate and cultured in Schneider’s Insect Medium supplemented with 20% FBS and 1× antibiotic/antimycotic overnight at 32 °C. Hemocytes were then exposed to 0.8, 8, and 80 μM chlorothalonil or solvent control (0.1% DMSO) for 16 h. The effects on mitochondrial bioenergetics (basal respiration and spare capacity) in the presence of glutamate were evaluated by perturbing hemocytes with a series of model mitochondrial toxicants and ATP-dependent, maximum, and proton-leak dependent respiration was measured (Mito Stress Kit, Agilent, Santa Clara, CA, USA). Significant effects on basal respiration and spare capacity were observed at 8 and 80 μM. These results suggest that exposure to chlorothalonil could reduce respiration rates through increased production of reactive oxygen species and disruption of the mitochondrial membrane potential, although the mechanisms underlying observed changes are not well understood. Additional studies are underway to determine the effects of chlorothalonil on immune function of honey bee hemocytes with the goal of understanding impacts this fungicide may have on honey bee health.

### 2.34. Investigating the Effects of Fungicides on Honey Bee Survivorship, Learning, and Physiology

NearmanAnthonyvanEngelsdorpDennisUniversity of Maryland, College Park, MD, USA

Honey bees are required for much of the cucurbit pollination in the northeast and mid-Atlantic regions. Chlorothalonil is a common standard among conventional cucurbit growers in these regions but is also known to negatively impact honey bee health. As part of a larger effort to provide cucurbit farmers with knowledgeable recommendations on fungicide use, we compared the effects of various field-relevant doses of fungicides (boscalid-pyraclostrobin mix and chlorothalonil) to bees with no exposure. Previous field experiments in this larger effort have revealed the levels at which the active ingredients in the selected fungicides persist in watermelon pollen. In our current study, these measurements were used to dope pollen substitute with the same formulas used in those field experiments. The doped pollen substitute was then analyzed by the same methods used to analyze the watermelon pollen (to ensure effective doping procedures) and subsequently provided to bees in a series of cage experiments. The initial set of experiments involved multiple replicates of 4 treatment groups (two, separate levels of doping per fungicide) compared to a control (non-doped pollen substitute) where mortality was tracked until all bees had died. A second set of cage experiments was executed to determine the effects of fungicides on learning by measuring the Proboscis Extension Reflex and Retention. Bees were caged for a period of two weeks while continuously offered pollen substitute, in the same arrangement as the survivorship experiment, and then tested on their ability to learn and retain that learning. Last, bees were again caged in the same arrangement of treatment vs control but continually harvested for autopsy over the course of twenty days. Five bees per cage were harvested over six time points and then autopsied to document and compare 19 different physiological traits noted by previously published work as potential indicators of honey bee health. Additional methods of this effort are still in development and involve microinjection of a controlled inoculum for measure of sub-lethal effects. Current analysis indicates the presence of some treatment effects. Furthermore, this suite of methods may provide an outline for future testing of other honey bee stressors.

### 2.35. Diamide Modulation of the Ryanodine Receptor in a Beneficial and Pest Insect Species

WilliamsJennifer R.SwaleDaniel R.AndersonTroy D.Department of Entomology, University of Nebraska, Lincoln, NE, USA

Honey bee decline is a nationally-recognized problem that demands attention from both the scientific community and beekeeping industry. One outstanding threat is the unintended exposure of these pollinators to agricultural pesticides. The ryanodine receptor (RyR) is a ligand gated calcium channel found in cardiac, neuronal, and muscle cells which mitigates calcium flow for muscle contractions. Emphasis on this underutilized target site led to the discovery of a novel insecticide class, the diamides. Many studies regarding efficacy of diamides report their selectivity for lepidopteran or hemipteran pests over beneficial arthropods, but do not elucidate on the possible mechanisms for this increased efficacy. There are few publicly available studies that provide an in-depth analysis of diamide effects on beneficial insect pollinators, such as honey bees. This study focuses on diamide insecticide exposures to honey bees, a crop pollinating insect, and the fall armyworm, a crop pest insect. The data gathered will provide the relative acute toxicity, metabolic detoxification strategies, behavioral fatigue responses, and RyR modulator manipulation for bees and fall armyworms exposed to three current formulations of chlorantraniliprole. The future directions of this study will be discussed with emphasis on RyR modulation effects on muscle contraction and calcium mobilization, in addition to effects on thermoregulation in bees. This mechanistic study presents a top-down approach to examine diamide insecticide exposures to a beneficial species at the cellular, organ, and organismal levels and, in turn, the gathered data will serve to bridge knowledge gaps related to diamide insecticide exposures and pollinator health.

### 2.36. Characterization of Synergistic Toxicity in Pesticide Combinations on Honey Bees

WalkerEmily K.JohnsonReed M.Dr.LeslieBen J.Dr.Department of Entomology, The Ohio State University, Wooster, OH, USA

Honey bees are major pollinators and are crucial for both the survival of the agriculture industry and maintenance of the ecosystem. One of the major crops pollinated by honey bees is the almond. This study focused on two pesticides that are applied to almonds in combination. This project concentrated on the spray application of these pesticides through the use of a Potter Tower, which is a laboratory apparatus used to study the biological effects of chemicals when applied as a spray. The two pesticides that were studied in this project were the fungicide Tilt (active ingredient propiconazole) and the insecticide Altacor (active ingredient chlorantraniliprole). These chemicals are not known to be toxic to bees when applied separately, but have shown synergistic toxicity when combined. The chemical Altacor acts to paralyze insects, but this toxicity is not observed with honey bees, even at high concentrations. The lack of toxicity of Altacor in honey bees is believed to be caused by the rapid metabolism of the pesticide before it can cause paralysis. It was hypothesized that the combination of Tilt and Altacor is toxic to honey bees because Tilt inhibits the cytochrome P450 enzyme(s) that breaks down Altacor, which allows Altacor to paralyze and kill the honey bees. This hypothesis was supported by similar synergistic toxicity when a known cytochrome P450 inhibitor, Piperonyl Butoxide (PBO), was combined with Altacor and applied to honey bees using the Potter Tower. Additional research will be conducted using LC-MS to further characterize the metabolic effects of the Tilt and Altacor combination on the honey bee. This study suggests that the pesticides Tilt and Altacor, which are commonly applied to almonds, are toxic in combination and may be a possible explanation for large numbers of honey bee losses.

### 2.37. Differences in Larval Pesticide Toxicity across Honey Bee (Apis mellifera) Stocks

MiloneJoseph P.TarpyDavid R.Department of Entomology & Plant Pathology, North Carolina State University, Raleigh, NC, USA

Honey bee (*Apis mellifera*) colonies accumulate chemical residues in wax and stored food, which exposes brood to multiple pesticides during sensitive developmental stages. However, the harmful effects of these exposures depends on the sensitivity of bees’ to a given dose. We compared pesticide susceptibility among seven honey bee breeding stocks using a larval oral exposure assay (Figure 5). To test field-realistic exposures, we selected a mixture of seven commonly detected insecticides, fungicides, and an herbicide using previously reported pesticide residue data from commercial colonies. We established queens from each stock into full sized colonies which were then used as larval grafting sources (see figure). We chronically administered a diet spiked with the treatment mixture at four doses during worker larval development *in vitro*. We calculated a hazard quotient (HQ) in order to quantify the toxicity of the multi-pesticide mixture at each concentration and compared survivorship to the exposure among the offspring from 28 queens. When comparing median lethal HQs we found a gradient of pesticide sensitivity across stocks. We report that those larvae from putatively feral and more pure old world stocks were the most tolerant to pesticide exposure. Alternatively, larvae from a lineage which has been highly selected for hygienic behavior against the varroa mite were found to be the most susceptible to our experimental multi-pesticide treatment mixture. These findings highlight the impacts of artificial selection and how unintended consequences may occur during systematic breeding for specific traits in honey bees. Additionally, more work is needed to uncover the mechanism driving differential larval pesticide tolerance across stocks.

### 2.38. Impact of Chronic Dietary Thiamethoxam and Prothioconazole Exposure on Survival of Apis mellifera L. Brood and Adults Reared In Vitro

WoodSarah C.Department of Veterinary Pathology, Western College of Veterinary Medicine, University of Saskatchewan, Saskatoon, SK, Canada

Results and discussion were presented regarding the following now-published manuscript:

Wood, S.C.; de Mattos, I.M.; Kozii, I.V.; Klein, C.D.; Dvylyuk, I.; Folkes, C.D.; de Carvalho Macedo Silva, R.; Moshynskyy, I.; Epp, T.; Simko, E. Effects of chronic dietary thiamethoxam and prothioconazole exposure on *Apis mellifera* worker adults and brood. *Pest Manag. Sci.* **2019**, *76*, 85–94. doi:10.1002/ps.5501.

### 2.39. Reproductive Fitness of Honey Bee Queens Exposed to Thiamethoxam during Development

KoziiIvanna V.WoodSarah C.KleinColby D.SilvaRoney de C.M.FabelaClaudia I.O.FolkesCrystaniDvylyukIhorde MattosIgor M.GuilleminLelandSimkoElemirWestern College of Veterinary Medicine, University of Saskatchewan, Saskatoon, SK, Canada

Productivity and survival of the honey bee colony depend on the reproductive potential and health status of the honey bee queen. Poor queen quality has been reported as one of the main reasons of colony losses. Several studies have shown that neonicotinoid pesticides negatively affect reproductive potential of honey bee queens; however, the mechanisms and extent of this effect are not well understood. The aim of this study was to investigate the reproductive fitness of queens exposed to neonicotinoid thiamethoxam (THI) during larval and pupal development. The queens used in this study were reared from 3.5 day old larvae in queen-right colonies following standard beekeeping procedures. At day 7 post oviposition each queen larva was exposed to 4ul of distilled water containing 0 ng (control), 5 ng or 50 ng of THI (N = 47, 51, 60 queens respectively). The test solution was directly pipetted into the royal jelly of each queen cell with subsequent monitoring of larval/pupal survival. At day 14 of development exposed queen cells were introduced to 3-frame nucleus colonies. Mated queens were collected 21 days post-emergence. Survival, total sperm count and viability were recorded. In addition, total mandibular gland area was assessed histologically based on serial head sections. Survival of queens until day 14 of development was 100% in the control group, while it decreased to 83% and 63% in low and high dose groups, respectively. No treatment effect was observed on total sperm count; however, sperm viability decreased in individuals exposed to 50 ng THI by 13% compared to control. Mandibular glands of queens exposed to 50 ng THI were 20% smaller than those in the control group. The effect of 5 ng of THI on mandibular glands is being investigated. Environmentally relevant doses (even at the highest estimated contamination levels of 5ng/larva) did not have a significant effect on sperm quality and pupal survival. Detectable effects were observed when the doses were increased 10 times from the highest estimated environmental contamination.

### 2.40. The Effects of Agrochemicals on Honey Bee (Apis mellifera) Queen Mating Frequency and Sperm Viability

WalshElizabethJanowieckiMarkVargoEdwardRangelJulianaDepartment of Entomology, Texas A&M University, College Station, TX, USA

Multiple factors continue to affect honey bee (*Apis mellifera*, L.) health decline, including exposure to pesticides and the globally present ectoparasite, the varroa mite (*Varroa destructor*). Honey bee colonies can be exposed to beekeeper-applied miticides such as amitraz, coumaphos, and tau-fluvalinate, or field-applied pesticides such as chlorothalonil and chlorpyrifos, which are encountered on crops by forager bees; all these pesticides are among the top 10 found in surveys of beeswax comb ubiquitously found throughout the U.S (Mullin et al. 2010). Here we explored whether pesticide contamination of the beeswax used by workers for queen rearing affects the reproductive health of adult queens. We reared queens in cups coated with either pesticide-free wax or wax contaminated with field-relevant concentrations of (1) amitraz, (2) a combination of coumaphos and tau-fluvalinate, or (3) a combination of chlorothalonil and chlorpyrifos. After queens emerged, they were placed in queenless mating nucleus colonies and allowed to mate naturally. We measured sperm viability and the mating frequency of the resulting mated queens. Sperm viability was assessed using dual fluorescent microscopy (spermatozoa were differentially stained with SYBRGREEN if viable and Propidium Iodide if non-viable) and a cell counter. Mating frequency was assessed using 8 different microsatellites in worker pupae offspring of the queens in question. Sperm viability of the different queens was apparently not impacted by experimental group, but mating frequency was different between control and some experimental groups. Our results imply that beekeeper- and field-applied pesticides directly affect queen reproductive health and are thus vital for the apicultural industry to know.

### 2.41. Potential Interaction Effects: How Do Parasitic Mites and Neonicotinoid Insecticides Simultaneously Affect Honey Bee Food Glands?

BrucknerSelinaWilliamsGeoffrey R.Department of Entomology & Plant Pathology, Auburn University, Auburn, AL, USA

Neonicotinoid insecticides and the ectoparasitic mite *Varroa destructor* are known to independently cause lethal and sub-lethal effects in honey bees. Despite the importance of both stressors to honey bees, as well as their ubiquitous nature, few have investigated the effects of simultaneous exposure. Recent studies demonstrated negative effects of neonicotinoids on nursing worker hypopharyngeal glands (HPGs). Because of the importance of HPGs to brood food production for all honey bee types (queen, worker, drone), we examined the effects of simultaneous neonicotinoid and *V. destructor* exposure on HPGs by performing a fully crossed experimental design. We obtained known age cohorts of worker honey bees from 24 colonies which were previously fed with pollen patties for 49 days. Half the colonies received patties that contained field-realistic concentrations of neonicotinoids (3.25 ppb thiamethoxam), whereas the other half received patties without neonicotinoids. Workers from each colony were artificially emerged, assessed for *V. destructor* parasitism, and allocated to one of four treatments: (1) No neonicotinoid/No *V. destructor*, (2) No neonicotinoid/Yes *V. destructor*, (3) Yes neonicotinoid/No *V. destructor*, and (4) Yes neonicotinoid/Yes *V. destructor*. Workers were maintained in laboratory cages with sugar syrup and pollen patties for 10 days, the typical age of nursing, before being decapitated for HPG examination. Here we discuss the effects of neonicotinoid insecticides and *V. destructor* parasitism, alone and in combination, on HPG size.

### 2.42. Honey Bee Workers Have a Critical Size (Poster)

LiHonghongHuangZachary Y.Department of Entomology, Michigan State University, East Lansing, MI, USA

Critical size is defined as the minimal weight at which additional food and further growth were not necessary for a normal time course to metamorphosis. However, Metamorphosis of a solitary bee species was induced by starvation rather than a critical size. As the most critical pollinator, honey bee plays a very important role in agricultural production and the entire ecosystem. We determined whether honey bee workers show a lack of critical size as in the solitary bees, or whether they do have a critical size. We removed worker larvae at different sizes and then recorded their time of pupation by infrared cameras. We found that honey bees do have a critical size, which ranged from 80 to 100 mg in five different trials. The implication of this finding will be discussed.

### 2.43. Physiological Mechanisms of Fitness Deficits in Capped Brood Apis mellifera Exposed to Critical Temperatures (Poster)

XuXinjianHaoZhenbangZhuXiangjieZhouShujingZhouBingfengDepartment of Entomology, Michigan State University, East Lansing, MI, USA

The honeybee brood was stenothermic and critical thermal exposure imposed detrimental effects on their development fitness. To update little is known on the physiological mechanism underlying thermal effects on bee brood development fitness. More than 80% brood could not emerge at 29 °C while none could survived at 38 °C. Capped brood developmental duration deceased as the temperature increasing from 29–37 °C; The emerged pupae energy reserve at 29 °C was the least among the test temperatures due to an extended duration which may explain their high mortality. while the total energy use was accumulated in unfulfilled brood at 38°C. Capped brood exposure at 29 °C and 38 °C accumulated severe cell death in Malpighian tubes. Moreover, the carbonyl concentration was significantly increased in capped brood exposed to 29 °C and 38 °C. Our data explored the critical temperatures affecting the energy use during honeybee capped brood development, which provides insights into physiological mechanism underlying the stenothermic requirements for honeybee brood.

### 2.44. The microRNA ame-miR-279a Regulates Sucrose Responsiveness of Forager Honey Bees (Apis mellifera) (Poster)

LiuFangShiTengfeiYinWeiSuXinQiLeiHuangZachary Y.ZhangShaowuYuLinshengDepartment of Entomology, Michigan State University. East Lansing, MI, USA

Increasing evidence demonstrates that microRNAs (miRNA) play an important role in the regulation of animal behaviours. Honey bees (*Apis mellifera*) are eusocial insects, with honey bee workers displaying age-dependent behavioural maturation. Many different miRNAs have been implicated in the change of behaviours in honey bees and ame-miR-279a was previously shown to be more highly expressed in nurse bee heads than in those of foragers. However, it was not clear whether this difference in expression was associated with age or task performance. Here we show that ame-miR-279a shows significantly higher expression in the brains of nurse bees relative to forager bees regardless of their ages, and that ame-miR-279a is primarily localized in the Kenyon cells of the mushroom body in both foragers and nurses. Overexpression of ame-miR-279a attenuates the sucrose responsiveness of foragers, while its absence enhances their sucrose responsiveness. Lastly, we determined that ame-miR-279a directly target the mRNA of Mblk-1. These findings suggest that ame-miR-279a plays important roles in regulating honey bee division of labour.

### 2.45. Investigating Potential Impacts of Pesticide Residue Accumulation on Varroa Mite Fecundity and Colony Health (Poster)

AlbrechtJenniferWu-SmartJudyDepartment of Entomology, University of Nebraska, Lincoln, NE, USA

Honey bees (*Apis mellifera* L.) continue to struggle worldwide due to factors such as pests, pathogens, pesticides, and poor nutrition, that may act alone or in combination with other stressors. Pesticide accumulation in comb and food stores can prevent colonies from producing healthy queens, increase susceptibility to pests and pathogens, and cause other adverse effects on over-wintering success. For example, previous studies indicate brood development may become delayed when bee larvae are reared in pesticide-laden comb. Delays in development time may provide a reproductive advantage to varroa mites (*Varroa destructor*) a major pest that causes issues including virus transmission, deformities, and reduced colony strength. To examine the potential interactions between pesticide exposure and mite infestation, worker bees were reared in clean versus pesticide-laden comb. Bee mortality and development time were monitored from egg to adult emergence. During the pre-pupal stage, host brood cells were artificially infested with mites. *Varroa* fecundity along with bee health measures, such as hypopharyngeal gland size and longevity, were evaluated across treatments. Pesticide accumulation is a consistent problem but effects on bee health and methods to mitigate pesticide accumulation in hive products have not been well-studied. The use of dead bee traps for proactive pesticide incident monitoring and alternative comb replacement options to provide beekeepers more economic solutions to mitigate pesticide accumulation will also be discussed. Results from this study will be of particular importance in determining how major factors, such as pesticides and mites, may interact and synergize adverse effects that contribute to colony decline.

### 2.46. Exploring the Effects of Pesticide Residues in Brood Comb on the Behaviors and Development of the Honey Bee (Poster)

McDanielsWhitneyWu-SmartJudyDepartment of Entomology, University of Nebraska, Lincoln, NE, USA

Globally, there are 2.5 million tons of pesticides applied each year, and the United States alone uses more than 1.5 billion pounds of pesticides per year. When pesticides are applied, they may contaminate the nectar or pollen of the plants, which can then cause foraging bees to unknowingly bring the pesticides into the hive. This may be deposited into wax comb, and laying queens then deposit eggs in this comb which subjects the brood to varying concentrations of pesticide residues. The purpose of this project was to observe the behavioral and developmental effects of this pesticide-laden environment by asking the questions: will high levels of pesticide residues in brood comb cause (1) temporal shift in development time, age-related division of labor, and or longevity in bees exposed during larval development (2) adult bees (exposed during larval development) to exhibit lower hygienic behavior within the colony? To conduct this study, blocks of dirty and clean combs were paired together and placed in a common hive. A push-in cage was placed over the paired blocks of comb with a mature queen to allow the queen to lay eggs in the designated brood comb. Once the bees from each treatment emerged, they were tagged with a colored and numbered queen tag and were then transferred to an observation hive. Daily observations were taken to monitor normal development and participation in temporal polyethism. Adult bees were also tasked with performing hygienic behavior via freeze-killed brood to compare among treatment groups.

### 2.47. Exploring the Effects of Old Brood Comb on Larval Honey Bee (Apis mellifera L.) Survival (Poster)

MurrayStephanie K.JohnsonReed M.The Ohio State University, Columbus, OH, USA

Beeswax is the substrate on which every hive activity is performed. After years of life-sustaining activities, wax brood combs become darker and thicker, as they are layered with larval excrement, silken cocoons, and propolis. Unfortunately, brood comb can also act as a sink for pesticide residues and harmful microbes. This heavy use and potential for contamination has led beekeepers to practice brood comb replacement—replacing old combs with fresh plastic or wax foundation after several years of use. Brood comb replacement was introduced to improve the health and survival of European honey bee (*Apis mellifera* L.) colonies, however, a national survey of North American beekeepers has shown otherwise. Survey results are ambiguous, but often point to better survival on old brood comb. To provide beekeepers with accurate brood comb management tools, we will begin by measuring larval survival on combs of different ages. More than twenty packages of bees were randomly installed on clean or heavily used wax treatments, and larval survival was measured using a modified Liebfelder method. The results of this study will demonstrate that heavily used brood comb is either a detriment, a benefit, or has no effect on the survival of *A. mellifera* larvae. Regardless of the outcome, this study will provide information applicable to honey bee survival; beekeepers can either manage their brood comb accordingly, or put their money and energy into more effective management tactics.

### 2.48. Using a Multi-Omics Approach to Understand Bee Nutritional Landscape

ChakrabartiPriyadarshini^1^MorréJeffery T.^2^LucasHannah M.^1^MaierClaudia S.^2^SagiliRamesh R.^1^1Department of Horticulture, Oregon State University, Corvallis, OR 97331, USA2Department of Chemistry, Oregon State University, Corvallis, OR 97331, USA

Poor nutrition has been cited as one of the major factors involved in honey bee declines that have been reported over the last decade. Since reports of significant bee declines began, much effort has been dedicated to researching the causes of such declines, but only a few studies have addressed the underlying, fundamental problems, particularly with regard to nutrition. As honey bee nutrition plays a vital role in mitigating the effects of biotic and abiotic stressors on bees, endeavoring to improve bee nutrition is critical. Phytosterols, especially 24-methylenecholesterol, play an important role in bee physiology. It is important to understand which dietary sources provide important phytosterols for all bee species. Thus, a robust method has been developed to identify major phytosterols from various dietary sources for honey bees through a targeted lipidomics approach using mass spectrometry. Liquid chromatography/Mass spectrometry methods helped study the major phytosterol profiles (Figure 6) as well as the important metabolites (Figure 7) across a wide spectrum of bee dietary sources—plant pollens, commercial diet and vegetable oils. 

It was observed that each dietary sample type differed in its sterol composition with 24-methylenecholesterol being found in trace amounts in the commercial diet and present in its highest abundance in the pollen samples, followed by the vegetable oils. Important metabolites, such as vital amino acids, flavonoids, phenolic acids etc. were all differentially detected across the various dietary samples. The implications of these findings will help formulate feasible practical recommendations for improving honey bee health by providing optimal nutritional sources. The details of the study can be found in Chakrabarti, P., Morré, J.T., Lucas, H.M., Maier, C.S. and Sagili, R.R. The omics approach to bee nutritional landscape. *Metabolomics* **2019**, *15*, 127. doi:10.1007/s11306-019-1590-6.

## Figures and Tables

**Figure 1:**
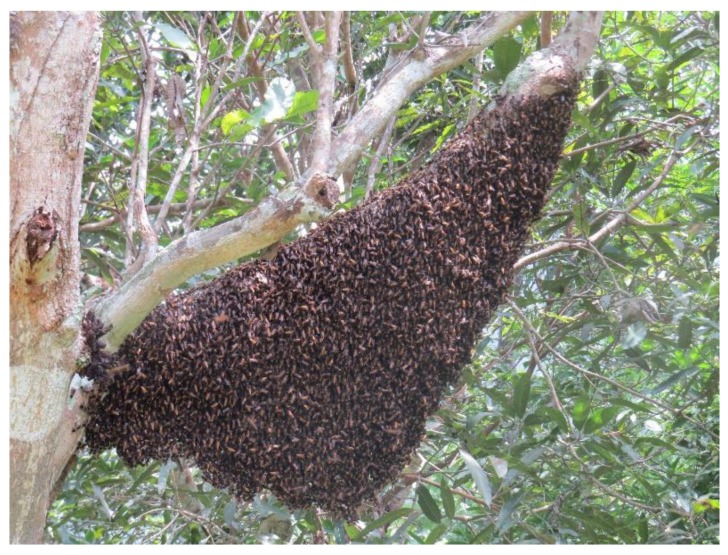
A large bivouac of giant honey bees, still agitated from its arrival flight, settles on a mango branch at the study site. Photo: Willard S. Robinson.

**Figure 2:**
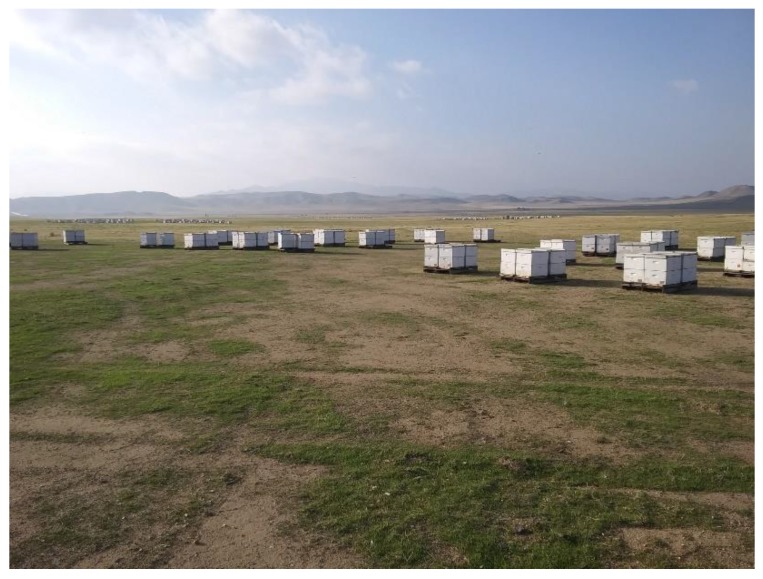
Experimental colonies used in the study in a holding yard, prior to almond pollination, California.

**Figure 3:**
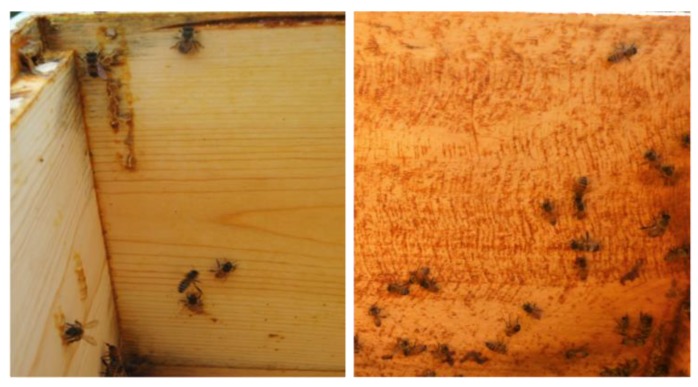
Conventional smooth box with propolis placement in corners and at frame rests (left) vs. the roughened box interior with a more continuous distribution of propolis.

**Figure 4:**
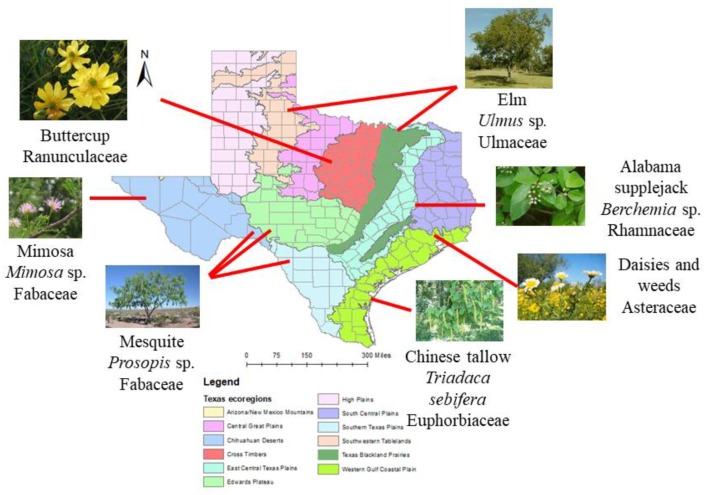
Notable taxa detected in honey from different ecoregions in Texas within Texas.

**Figure 5:**
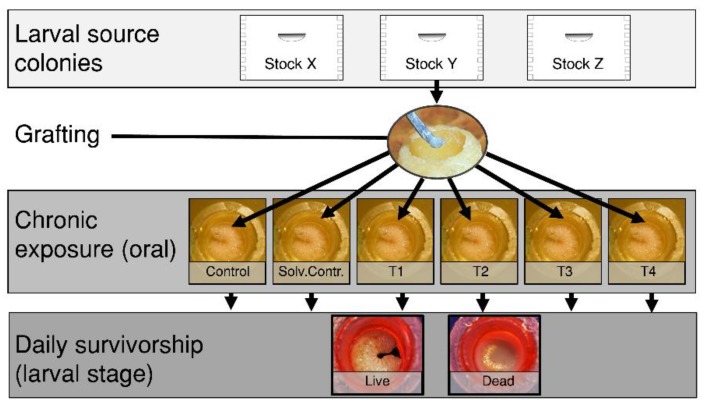
Graphical representation of methods.

**Figure 6:**
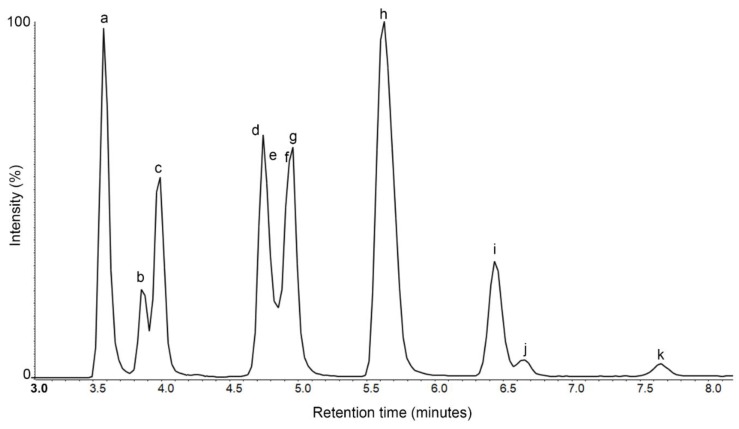
Reproduced from Chakrabarti, P.; Morré, J.T.; Lucas, H.M.; Maier, C.S. and Sagili, R.R. The omics approach to bee nutritional landscape. *Metabolomics* **2019**, *15*, 127, doi:10.1007/s11306-019-1590-6. The figure represents a total ion chromatograph of a representative sterol standard mix (75 μm). The sterol peaks identified are as follows: (**a**) desmosterol; (**b**) ergosterol; (**c**) 24-methylenecholesterol; (**d**) stigmasterol; (**e**) brassicasterol; (**f**) Δ^5^-avenasterol; (**g**) cholesterol; (**h**) campesterol; (**i**) β-sitosterol; (**j**) campestanol and (**k**) sitostanol.

**Figure 7:**
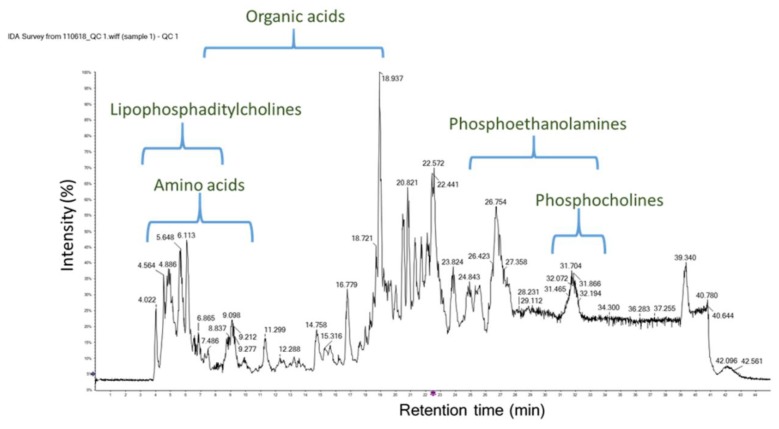
Reproduced from Chakrabarti, P.; Morré, J.T.; Lucas, H.M.; Maier, C.S. and Sagili, R.R. The omics approach to bee nutritional landscape. *Metabolomics* **2019**, *15*, 127, doi:10.1007/s11306-019-1590-6. The figure represents a total ion chromatograph of a representative metabolomics study, highlighting a few major groups of compounds identified across various retention times.

